# AI technology adoption and intergenerational knowledge transfer among older employees

**DOI:** 10.3389/fpsyg.2025.1673730

**Published:** 2025-11-26

**Authors:** Yanhong Guo, Li Wei

**Affiliations:** School of Economic Management and Law, Jilin Normal University, Siping, China

**Keywords:** AI technology adoption, intergenerational knowledge transfer, relational crafting, older employees, identity threat

## Abstract

**Introduction:**

This study employs the Job–Demands-Resources model and Conservation of Resources theory to examine the impact of artificial intelligence (AI) technology adoption on intergenerational knowledge transfer among older employees. It focuses on the psychological motivation underlying this phenomenon and identifies individual factors that affect intergenerational knowledge transfer. The purpose is to gain a deep understanding of the internal mechanisms of employee cognition and behavior change in the context of technological transformation.

**Methods:**

We surveyed 635 older employees from various industries in China and analyzed the data using SPSS 27.0, Mplus 8.3, and fsQCA 4.1. The data were analyzed via a moderated sequential mediation model to examine the relationships among AI technology adoption, identity threat, relational crafting, digital self-efficacy and intergenerational knowledge transfer, supplemented by fuzzy-set qualitative comparative analysis (fsQCA). The study tested the mediating effects of identity threat and relational crafting between AI technology adoption and intergenerational knowledge transfer, as well as the moderating role of digital self-efficacy. In addition, fsQCA was used to test antecedents of intergenerational knowledge transfer among older employees.

**Results:**

The findings indicate that AI technology adoption positively influences intergenerational knowledge transfer. Identity threat and relational crafting play mediating roles between AI technology adoption and intergenerational knowledge transfer and also serve as sequential mediators. Digital self-efficacy negatively moderates the impact of AI technology adoption on identity threat, thereby moderating both the mediating role of identity threat and the sequential mediating effect of identity threat and relational crafting. Additionally, fsQCA identified three antecedent configurations that trigger intergenerational knowledge transfer among older employees.

**Discussion:**

Prior research on AI technology adoption has tended to emphasize singular positive or negative impacts on specific variables. This study constructs a model that incorporates both positive and negative effects, elucidating the multifaceted mechanisms through which AI technology adoption influences intergenerational knowledge transfer and enriches research on the consequences of AI technology adoption. While existing literature often highlights negative psychological and behavioral impacts of AI technology adoption on older employees, the present findings show that AI technology adoption can significantly enhance intergenerational knowledge transfer among older employees, thereby complementing current findings. Finally, by adopting a configurational thinking, this study identifies multiple pathways through which various factors affect intergenerational knowledge transfer, providing a useful complement to single-factor analyses of AI technology adoption’s impact. Thereby, the study offers practical insights for organizations seeking to develop inclusive technological-culture strategies.

## 1 Introduction

For Artificial Intelligence (AI) serves as a vital tool for the transformation and upgrading of enterprises worldwide, enabling effective knowledge creation, standardization, and sharing, thereby enhancing organizational competitiveness. At the same time, organizations are increasingly adopting AI technology as a key means to reduce costs, improve customer satisfaction, and strengthen product competitiveness (Mariani and Borghi, 2021). This trend is challenging the foundations of traditional organizational operations and transforming employees’ work environments, processes, and tasks (Braganza et al., 2021; Tang et al., 2022). As a result, AI technology is profoundly influencing employee psychology and behavior (Dong et al., 2025), a phenomenon that has attracted widespread attention in both practical and academic. However, research on the psychological and behavioral effects of AI technology adoption on employees remains limited, particularly with respect to older employees. Due to the limitations of “digital immigration,” older employees are more likely to encounter difficulties when adopting AI technology (Román-García et al., 2016). This can trigger resistance, a sense of threat (Xu and Xue, 2023), and corresponding negative behaviors. Given the aging of the global labor market, older employees have already assumed critical positions and constitute a significant demographic group. As important carriers of organizational experience and knowledge, older employees directly affect the continuity and innovation ability of organizational experience and knowledge. In this context, how to ensure intergenerational knowledge transfer from older employees and prevent the disruption of knowledge and experience inheritance has become an unavoidable practical challenge in the intelligent transformation of organizations. What’s more, older employees show contradictory traits when it comes to adopting AI technology. On one hand, many in this group feel apprehensive or even threatened by AI technology. On the other hand, a significant number are actively learning and adapting to these new tools. It is worth noting that there are significant individual differences in the willingness to accept technology, adaptability, and behavioral performance among different older employees. These variations add another layer of complexity to management. Therefore it’s essential to bring these contradictions into clearer focus through research and to uncover the underlying logic and mechanisms of how older employees adopt AI, in order to fully depict the overall situation of AI technology adoption in organizations and make positive responses to existing management practices.

A review of existing research on AI technology adoption reveals mixed effects. On the positive side, under certain conditions, it can foster a cooperative atmosphere, improve employee morale (Qiu et al., 2022), and promote knowledge-sharing behaviors (Shaikh et al., 2023; Hu et al., 2025). On the negative side, it can generate perceptions of technological threat, insecurity and job risk (Brougham and Haar, 2020; Wu et al., 2024). Overall, these findings suggest that AI technology adoption may have either positive or negative impacts (Amisha et al., 2021), depending on the outcome variables examined. However, few studies have provided an integrated perspective to determine whether the same variable might simultaneously exert both positive and negative effects. Furthermore, existing research on the impact of AI technology adoption has yielded contradictory conclusions. For example, some scholars have found that AI technology adoption positively influences job satisfaction (Lestari et al., 2023), while others report the opposite (Nguyen and Malik, 2022). This raises a critical question: Are the inconsistencies due to methodological limitations in current research, or does AI technology adoption objectively produce two opposing effects on the same outcome variable? If this question remains unresolved, the inconsistencies in the literature will persist.

The application of AI necessitates organizational intelligentization to accelerate the iteration and renewal of existing technologies and knowledge (Liu et al., 2025). This shift alters the efficiency, pathways, and frequency of intergenerational knowledge transfer (Liu et al., 2020; Iaia et al., 2024). Consequently, investigating intergenerational knowledge transfer in the context of AI technology adoption has become imperative. This study therefore explores its potential positive and negative impacts on intergenerational knowledge transfer among older employees, as well as the underlying mechanisms. Moreover, AI technology adoption exerts both gain and loss pathways on employees. From the loss perspective, it induces a sense of identity threat and enhances perceived uncertainty about future work (Mirbabaie et al., 2022). From the gain perspective, it restructures social networks and fosters the establishment of new trust relationships (Dima et al., 2024). Thus, this study incorporates identity threat and relational crafting as mediating variables. Individual cognitive differences also lead to varied outcomes of AI technology adoption (Lazarus and Folkman, 1987). Employees with high digital self-efficacy adapt more effectively to changes brought about by AI (Maran et al., 2022). Accordingly, this study positions digital self-efficacy as a moderating variable.

In summary, this study focuses on the older employees, offering a meaningful response to workforce aging and its associated challenges. It provides a more realistic interpretation of management practices within organizational contexts, thereby enriching existing research. Crucially, it challenges the notion that older employees, as “digital immigrants,” can only face obstacles in adopting AI technology. Instead, it clarifies the actual impact of AI technology adoption on this specific group, contributing to a more systematic understanding of AI technology adoption research. This study examined the relationship between AI technology adoption and intergenerational knowledge transfer, addressing the issue in existing literature where research on antecedents is abundant while studies on outcome variables remain insufficient. Furthermore, rather than following the conventional approach of applying the Technology Acceptance Model (TAM), this research systematically investigated the consequences of AI technology adoption using the Job Demands-Resources (JD-R) model and the Conservation of Resources (COR) theory. This shift not only broadens the theoretical foundations of AI technology adoption studies but also offers a more appropriate and precise framework for explaining its underlying mechanisms. In addition, by incorporating identity threat and relational crafting as mediators and digital self-efficacy as a moderator, the study constructed a moderated sequential mediation model that found coexisting positive and negative effects. This integrated perspective effectively demonstrates that AI technology adoption can simultaneously produce both types of effects on the same variable. It thus supplements earlier studies that reported only one-sided outcomes and helps resolve contradictions in existing findings. Finally, this study employed fsQCA to validate the proposed model. This approach compensates for the limitations of traditional linear analyses and offers deeper insight into the complexity and heterogeneity of older employees’ behaviors in the context of AI technology adoption.

## 2 Theoretical framework and development of hypotheses

### 2.1 Job demands–resources model

Job demands–resources model posits that all job characteristics can be categorized into job demands and job resources (Demerouti et al., 2001; Lewig et al., 2007; Shimazu and Schaufeli, 2009). Job demands generally refer to factors that require individuals to exert effort and incur costs to complete tasks, thereby depleting personal energy. By contrast, job resources are positive factors in the work environment. Excessively high job demands and insufficient resources are likely to trigger job burnout and negative outcomes, while ample resources enhance engagement and yield positive outcomes. Specifically, AI technology adoption can accelerate information flow and work efficiency, providing employees with new job and social resources. At the same time, it also imposes higher job demands, resulting in insecurity and role conflict. The coping hypothesis within the JD-R model suggests that employees in challenging environments (high demands) are better able to transform resources into performance. Under high demands, employees engage more fully in their work and acquire more resources (Lewig et al., 2007; Hakanen et al., 2008; Bakker, 2010), indicating that job demands and resources can interact under certain conditions. Therefore, it is necessary to consider comprehensively the simultaneous impact of new job demands and resources brought by AI technology adoption on older employees.

### 2.2 Conservation of resources theory

Conservation of resources theory posits that individuals strive to obtain, retain, cultivate, and protect resources they value. The impact of resource loss is greater than that of resource gain, and individuals must continually invest resources to prevent depletion. Particularly in situations of loss, replenishment and accumulation of resources become critical. Therefore, individuals take action to prevent loss (Halbesleben and Wheeler, 2011) and remain vigilant in conserving resources to manage demands and stress (Hobfoll, 2011). COR theory suggests that depending on environmental and individual characteristics, resources follow two distinct pathways: a gain spiral and a loss spiral. In other words, AI technology adoption can provide job resources, enhancing employees’ sense of gain and forming a gain spiral, while also imposing higher demands, creating a sense of loss and forming a loss spiral.

### 2.3 AI technology adoption and intergenerational knowledge transfer

Artificial intelligence technology refers to systems that correctly interpret external data and flexibly apply learning to perform tasks and achieve goals (Kaplan and Haenlein, 2019; Raisch and Krakowski, 2021). As research on AI technology adoption remains at a nascent stage, prior studies have mainly focused on antecedents (Ma and Huo, 2023; Norzelan et al., 2024). AI technology adoption is understood as the process whereby organizations introduce AI and delegate its use to individuals, encompassing both initial adoption intentions/behaviors (Khasawneh, 2008) and post-adoption continuance behaviors–including usage, routinization, and adaptation (Limayem and Cheung, 2007). Given AI’s broad applicability, this study adopts the latter definition: the sustained behaviors of individuals after organizations introduce AI and delegate its use. Regarding the generational classification, there is no unified academic standard. However, many studies use age 45 as the dividing line between younger and older employees (Kulik et al., 2016). In addition, cohorts born after 1980 are often defined as “digital natives,” as their formative years coincided with the rapid advances in computer science. Compared to “digital immigrants” (pre-1980), this group demonstrates greater acceptance of emerging digital technologies. This study adopts this classification. Research on intergenerational knowledge transfer typically defines it as knowledge exchange between significantly age-differentiated groups, including downward knowledge transfer from older to younger employees and upward transfer from younger to older employees (Noethen, 2011; Wang et al., 2017). Given the objectives of this study, we specifically focus on downward knowledge transfer.

According to COR theory, driven by the intrinsic motivation to conserve resources and adapt to external environments, older employees tend to transfer knowledge and experience to younger employees who are more adept at AI technology adoption. This transfer serves as a resource investment in exchange for future support from younger employees in learning AI. Moreover, AI itself constitutes a unique job resource, reshaping and optimizing knowledge exchange within organizations. In other words, both the intrinsic motivation of older employees and the resource properties of AI can enhance the frequency and efficiency of downward knowledge transfer. Based on this reasoning, we propose:

H1: AI technology adoption positively affects intergenerational knowledge transfer.

### 2.4 Mediating role of identity threat

Identity threat refers to the perceived risk that one’s distinctive attributes, values, or identity are undermined (Xiao and Bavel, 2012). With technological advances and their impact on work environments, Craig et al. (2019) proposed the concept of IT identity threat, wherein individuals experience diminished self-esteem and doubts about their value due to technology use. Milad et al. (2022) extended this concept to AI, defining AI-induced identity threat as a workplace-specific manifestation. AI technology adoption weakens traditional social networks and alters roles (Tang et al., 2022), leading older employees to experience identity threat. This depletes their physical and psychological resources. According to JD-R theory, such factors represent job demands. For older employees, long-established networks and processes are disrupted by AI, diminishing distinctiveness and control (Zlotowski et al., 2017), thereby heightening identity threat. In this state, they are more likely to adopt avoidance strategies (Pearsall et al., 2009), refusing to transfer knowledge to younger colleagues who are more adaptable to AI, as a way to preserve their organizational status. AI-induced turbulence is a key driver of knowledge hiding in the digital era (Arias-Pérez and Vélez-Jaramillo, 2022), while the identity threat arising from AI technology adoption continues to deplete the individual resources of older employees, thereby forming a loss spiral (COR theory). As a result, older employees cease to engage in downward intergenerational knowledge transfer. Based on this reasoning, we propose:

H2: Identity threat mediates the relationship between AI technology adoption and intergenerational knowledge transfer. AI technology adoption amplifies identity threat, thereby diminishing knowledge transfer.

### 2.5 Mediating role of relational crafting

Relational crafting, as a form of job crafting, refers to proactive actions individuals take to alter the quality and boundaries of their workplace relationships (Niessen et al., 2016). Within the JD-R model, relational crafting is the behavior of individuals actively taking measures to improve the quantity and quality of interactions with others, and is a typical work resource. AI technology adoption can reshape older employees’ perceptions of relationships and boundary (Perez et al., 2022), enhancing connections with younger employees. Relational crafting improves workers’ affective, normative, and continuance commitment, positively influencing organizational outcomes (Noesgaard and Jorgensen, 2024; Geldenhuys et al., 2020), thereby triggering more downward intergenerational knowledge transfer. Furthermore, AI-driven explicitation of tacit knowledge reconstructs the logic of knowledge sharing. Coupled with the relational crafting triggered by AI technology adoption, which can bring out new resources, and given that job resources inherently possess motivational characteristics, this fosters the sustained stimulation of older employees’ downward intergenerational knowledge transfer, forming a gain spiral (COR theory). Consequently, this study proposes the following hypothesis:

H3: Relational crafting mediates the relationship between AI technology adoption and intergenerational knowledge transfer. AI technology adoption enhances intergenerational knowledge transfer by strengthening relational crafting.

### 2.6 Sequential mediating roles of identity threat and relational crafting

The JD-R theory emphasizes that employees in challenging environments (high job demands) are better able to transform job resources into high levels of job performance. COR theory also indicates that in situations of resource loss, individuals invest resources to cope with threatening circumstances. When older employees perceive identity threat during AI technology adoption, this constitutes a high job demand situation. In response, older employees more actively mobilize their resources and engage in knowledge transfer to younger employees through relational crafting. In other words, while identity threat continuously depletes older employees’ physical and psychological resources, they may also adopt proactive strategies–such as building digital trust, bridging the digital divide, sharing experience, and demonstrating their irreplaceability within the organization–thereby expanding the boundaries and quality of their relationships. This process generates sufficient job resources and promotes increased downward intergenerational knowledge transfer. To summarize, this paper proposes the following hypothesis:

H4: Identity threat and relational crafting play sequential mediating roles between AI technology adoption and intergenerational knowledge transfer.

### 2.7 Moderating role of digital self-efficacy

Digital self-efficacy constitutes an extension of self-efficacy. Agarwal et al. (2000) define digital self-efficacy as an individual’s belief in their capability to effortlessly and effectively utilize information technology and adapt to digital devices, representing subjective perceptions when executing tasks or activities related to digital systems. According to COR theory, employees with different individual resources choose different ways to cope with AI technology adoption. Therefore, individuals with higher digital self-efficacy tend to show stronger adaptability and learning capability regarding AI technology. Compared to those with lower digital self-efficacy, they often possess more initial resources, experience less resource loss, and exhibit milder stress responses. In other words, individuals with higher digital self-efficacy are less likely to experience identity threat during AI technology adoption, consequently reducing the likelihood of resisting intergenerational knowledge transfer due to identity threat. Based on this reasoning, this study proposes:

H5: Digital self-efficacy negatively moderates the relationship between AI technology adoption and identity threat.

According to JD-R model, digital self-efficacy as an individual resource not only directly enhances work performance but also indirectly influences work engagement through pathways such as motivation enhancement and buffering effects (Bakker and Demerouti, 2017). Digital self-efficacy helps older employees to reassess available resources. Those with high digital self-efficacy tend to view AI technology as a supportive tool (Maran et al., 2022), effectively alleviating self-doubt regarding their competence and identity triggered by technological changes. Consequently, the defensive resource conservation motivation caused by identity threat is weakened (Arias-Pérez and Vélez-Jaramillo, 2022), which buffers the negative impact of identity threat on intergenerational knowledge transfer. In contrast, older employees with low digital self-efficacy often experience heightened identity threat due to lack of confidence, making them more likely to fall into resource depletion and activate defensive mechanisms, thereby hindering intergenerational knowledge transfer. In summary, digital self-efficacy effectively inhibits the initiation of the loss spiral from AI technology adoption to identity threat, indirectly mitigating the negative effect of identity threat on intergenerational knowledge transfer. Based on this, this study proposes:

H6: Digital self-efficacy positively moderates the indirect effect of AI technology adoption on intergenerational knowledge transfer through identity threat.

According to JD-R model, older employees with low digital self-efficacy exhibit a stronger motivation to conserve resources when faced with identity threat. To prevent further resource loss and restore resource equilibrium, they are objectively compelled to invest their resources into relational crafting. This enhances their influence in intergenerational relationships and indirectly promotes intergenerational knowledge transfer. In contrast, older employees with high digital self-efficacy, equipped with stronger technical adaptability and learning confidence, can more proactively adapt to technological changes and experience lower levels of identity threat. Consequently, they are more inclined to rely on their own capabilities rather than external pathways to adapt to technological transformations. That is, there is no need to achieve intergenerational knowledge transfer through relational crafting triggered by identity threats. Therefore, digital self-efficacy weakens this serial mediation path. Based on this reasoning, this study proposes:

H7: Digital self-efficacy negatively moderates the indirect effect of AI technology adoption on intergenerational knowledge transfer through the sequential mediation of identity threat and relational crafting.

**FIGURE 1:**
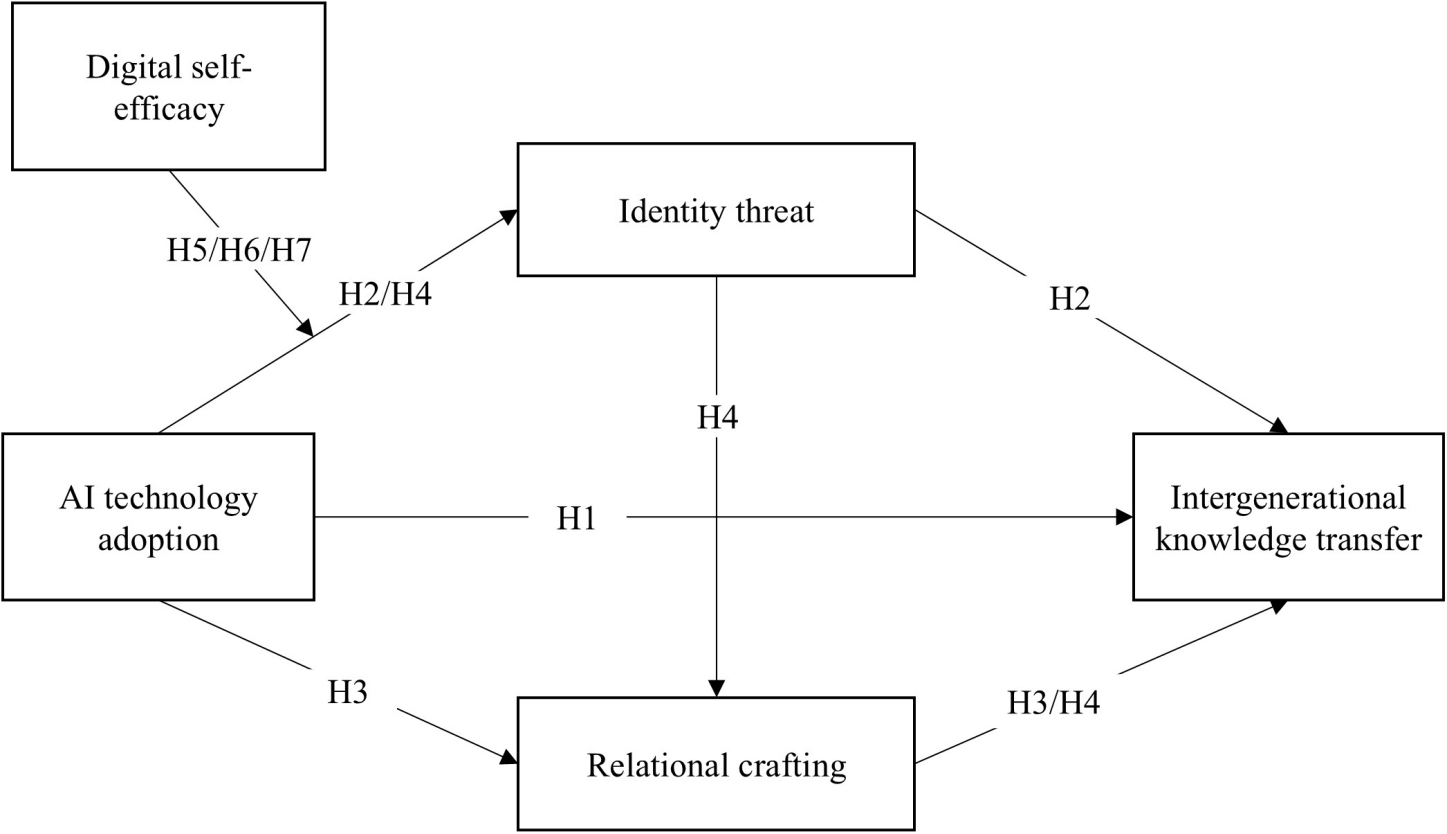
Theoretical model.

The theoretical model of this study is illustrated in Figure 1.

## 3 Materials and methods

### 3.1 Sample and procedure

This study focuses on older employees based on the following considerations. First, from a practical perspective, the global labor market is undergoing significant population aging, with older employees playing an increasingly critical role in organizations as key carriers of job experience and knowledge dissemination. However, amid corporate digital and intelligent transformation, the “digital divide” means that older employees–as “digital immigrants”–are more likely to face barriers in technological adaptation compared to their younger “digital native” employees. Such barriers may not only trigger negative psychological reactions (e.g., identity threat) but could also lead to the loss of valuable knowledge, experience, and organizational memory. Therefore, investigating the psychological and behavioral mechanisms of this specific group in the context of AI technology adoption has urgent practical significance. Second, from a theoretical perspective, focusing on older employees provides a unique and crucial context for examining JD-R model and COR theory in this study. For older employees, AI technology adoption may be perceived both job demand and job resource. In this context, the potential resource gains or losses experienced by older employees offer an ideal context for testing the proposed gain and loss pathways, as well as coping hypotheses of this research. In contrast, focusing solely on younger employees or using an age-diverse sample would obscure these mechanisms.

This study collected data through online questionnaire surveys. To ensure the authenticity and accuracy of the data, an anonymous response method was adopted, and screening questions were included: “Do you use AI technology on a regular basis in your work?” and “Are you over 45 years old?” These items ensured that participants were older employees who adopt AI technology. Furthermore, drawing on the approach of Tang et al. (2022), the definition of AI technology was provided in the survey instructions. After eliminating data with identical responses to consecutive items, outliers, inconsistent responses, and inattentive answers, 635 valid questionnaires were recovered, with a validity rate of 70.9%. In terms of gender, males accounted for 38.1% and females account for 61.9%; In terms of age, the sample was concentrated between 45 and 55 years old, accounting for 96.4%. Regarding education, 6.3% had junior high school or below, 10.6% had high school or vocational school, 9.3% had college degrees, 58.1% had undergraduate degrees, and 15.7% had master’s degrees or above, Regarding monthly income, 21.1% were below 4000 Yuan, 38.1% are between 4000 and 8000 Yuan, and 20% are between 8000 and 12000 Yuan, 9% are between 12000 and 16000 Yuan, and 11.8% is above 16000 Yuan; In terms of tenure in the unit, 34.8% of employees had served for less than 10 years, 44.9% have served for 10–20 years, 15.3% have served for 20–30 years, and 5% for 30 years or more.

### 3.2 Measures

To ensure the reliability and validity of the questionnaire, all scales used in this study were validated and employed a 5-point Likert-type response format ranging from 1 (“strongly disagree”) to 5 (“strongly agree”). AI technology adoption used the eight-items scale referenced by Dong et al. (2025), with example items such as “I need AI to help me do my work” and a Cronbach’s alpha of 0.913. Relational crafting adopted the five-item scale from Slemp and Vella-Brodrick (2014), with example items such as “Make an effort to get to know people well at work” and a Cronbach’s alpha of 0.839. Identity threat adopted the four-item scale from Yogeeswaran et al. (2016), Liu and Xie (2025), with example items such as “Technological advancements in the area of robotics are threatening to human uniqueness” and a Cronbach’s alpha of 0.868. Intergenerational knowledge transfer adopted the five-item scale from Bock et al. (2005), with example items such as “My knowledge sharing with other organizational members is good” and a Cronbach’s alpha of 0.716. Digital self-efficacy adopted the three-item scale from Kim et al. (2021), with example items such as “I think I can easily learn how to use digital devices” and a Cronbach’s alpha of 0.77. Additionally, gender, age, education, monthly income, and organizational tenure were selected as control variables based on prior research. Since education is a multicategorical variable, it was dummy-coded during hypothesis testing.

## 4 Results

### 4.1 Discriminant validity and confirmatory factor analysis

Using Mplus 8.3, confirmatory factor analysis was conducted to examine the discriminant validity among AI technology adoption, identity threat, relational crafting, intergenerational knowledge transfer, and digital self-efficacy. Results are presented in Table 1. Compared to alternative models, the five-factor model exhibited superior and acceptable fit indices (χ^2^ = 835.808, df = 265, χ^2^/df = 3.154, RMSEA = 0.054, SRMR = 0.054, CFI = 0.93, TLI = 0.921). These results confirm that the five variables are mutually independent and demonstrate good discriminant validity.

**TABLE 1:** Results of confirmatory factor analysis.

Model	χ^2^	df	χ^2^/df	RMSEA	SRMR	CFI	TLI
1. AIA, IT, RC, KT, DS	835.808	265	3.154	0.054	0.054	0.93	0.921
2. AIA, IT+RC, KT, DS	1999.121	269	7.432	0.097	0.094	0.789	0.765
3. AIA, IT, RC, KT+DS	1110.49	269	4.128	0.066	0.065	0.897	0.886
4. AIA, IT, RC+KT+DS	1346.821	272	4.952	0.075	0.066	0.869	0.755
5. AIA, IT+RC+KT+DS	2525.023	274	9.215	0.11	0.101	0.725	0.699
6. AIA+IT+RC+KT+DS	3754.603	275	13.653	0.137	0.122	0.576	0.537

*N* = 635. AIA, AI technology adoption; IT, identity threat; RC, relational crafting; KT, intergenerational knowledge transfer; DS, digital self-efficacy.

### 4.2 Common method variance

Since all independent, dependent, mediating, and moderating variables in this study were derived from self-reported data, potential common method bias was addressed using Harman’s single-factor test. An unrotated exploratory factor analysis incorporating all variables revealed five factors with eigenvalues greater than 1. The first factor accounted for 34.08% of the variance–below the 40% threshold and not exceeding 50% of the total variance explained–indicating no significant common method bias.

### 4.3 Descriptive statistics

Descriptive statistical analysis was performed using SPSS 27.0. Table 2 summarizes the means, standard deviations, and correlation coefficients for each variable. Results show that: AI technology adoption was significantly positively correlated with identity threat (*r* = 0.117, *p* < 0.01), relational crafting (*r* = 0.505, *p* < 0.01), and intergenerational knowledge transfer (*r* = 0.375, *p* < 0.01). Identity threat was significantly positively correlated with relational crafting (*r* = 0.196, *p* < 0.01) but significantly negatively correlated with intergenerational knowledge transfer (*r* = −0.08, *p* < 0.05). Relational crafting was significantly positively correlated with intergenerational knowledge transfer (*r* = 0.511, *p* < 0.01). These findings are consistent with the theoretical hypotheses. Variance Inflation Factor (VIF) tests for all variables were below 2, indicating low multicollinearity.

**TABLE 2:** Descriptive statistics and correlations among study variables.

Variable	M	SD	1	2	3	4	5	6	7	8	9	10
1. Age	1.206	0.553	1									
2. Gender	1.619	0.486	−0.088*	1								
3. Tenure	1.906	0.833	0.292**	−0.089*	1							
4. Income	2.523	1.250	0.177**	−0.144**	0.412**	1						
5. Education	3.665	1.062	−0.057	0.070	0.098*	0.404**	1					
6. AIA	3.953	0.762	−0.020	−0.030	−0.022	0.074	0.103**	1				
7. IT	3.367	0.933	−0.005	−0.046	−0.008	−0.002	−0.088*	0.117**	1			
8. RC	3.866	0.716	−0.035	−0.008	0.062	0.065	0.129**	0.505**	0.196**	1		
9. KT	3.731	0.677	−0.070	0.053	0.026	0.064	0.124**	0.375**	−0.080*	0.511**	1	
10. DS	3.854	0.700	−0.056	−0.028	0.034	0.097*	0.128**	0.524**	0.173**	0.557**	0.477**	1

*N* = 635; AIA, AI technology adoption; IT, identity threat; RC, relational crafting; KT, intergenerational knowledge transfer; DS, digital self-efficacy, the same below; *p < 0.05, **p < 0.01.

### 4.4 Hypothesis testing

#### 4.4.1 Direct and mediation effect testing

**FIGURE 2:**
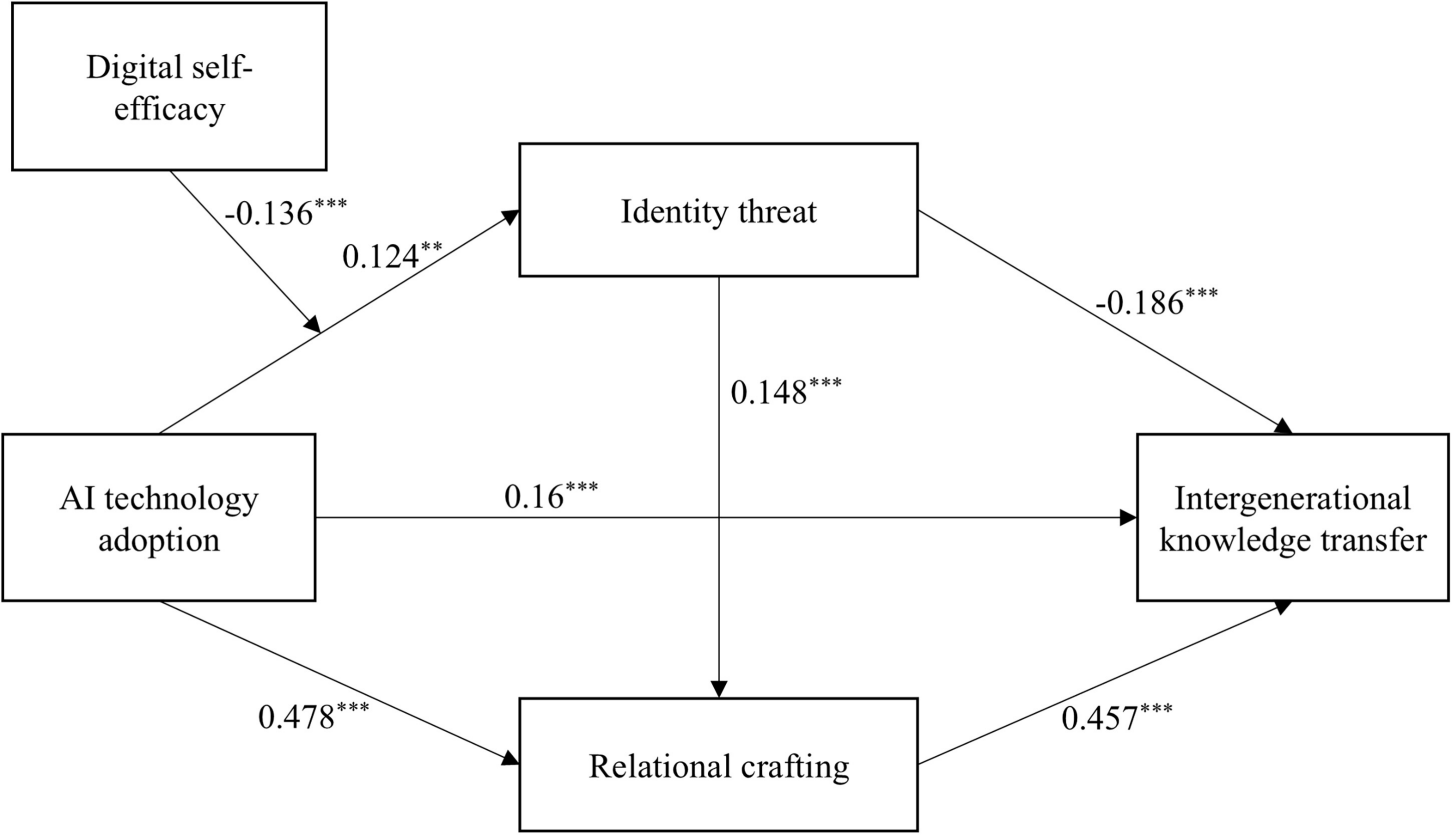
Results of path coefficient analysis. **p < 0.01, ***p < 0.001.

Analysis was performed using Model 6 in the PROCESS macro version 4.2. All variables were standardized, and the hypothesized model was tested using the bootstrap method with 5000 resamples, controlling for gender, age, tenure, education, and monthly income. Results (Table 3) show that: AI technology adoption significantly predicts intergenerational knowledge transfer (β = 0.16, *p* < 0.001), supporting Hypothesis 1. AI technology adoption significantly predicts identity threat (β = 0.124, *p* < 0.01). Identity threat significantly predicts intergenerational knowledge transfer (β = −0.186, *p* < 0.001). Further bootstrap analysis revealed that identity threat mediates the relationship between AI technology adoption and intergenerational knowledge transfer. The indirect effect was −0.023, 95% CI = [−0.041, −0.007], excluding zero, indicating a significant mediating effect. Hypothesis 2 is supported. AI technology adoption significantly predicts relational crafting (β = 0.478, *p* < 0.001). Relational crafting significantly predicts intergenerational knowledge transfer (β = 0.457, *p* < 0.001). Further comparison revealed that relational crafting mediates the relationship between AI technology adoption and intergenerational knowledge transfer. The indirect effect was 0.218, 95% CI = [0.165, 0.275], supporting Hypothesis 3. Additionally, integrating Table 3 findings with prior analysis indicates that: AI technology adoption positively influences identity threat. Identity threat positively influences relational crafting (β = 0.148, *p* < 0.001). Relational crafting positively influences intergenerational knowledge transfer. Results in Table 4 show that identity threat and relational crafting play sequential mediating roles between AI technology adoption and intergenerational knowledge transfer. The sequential indirect effect was 0.008, 95% CI = [0.002, 0.018], excluding zero, confirming the mediating effect. Hypothesis 4 is supported. The path analysis results are based on the sequential mediation model and the detection of the first-stage moderating effect, as shown in Figure 2.

**TABLE 3:** Regression results for main, mediation effects.

Variable	Model 1: IT		Model 2: RC		Model 3: KT	
	Value	SE	Value	SE	Value	SE
Gender	−0.028	0.041	0.006	0.035	0.053	0.034
Age	−0.014	0.042	−0.031	0.036	−0.049	0.035
Income	0.037	0.05	−0.013	0.043	0.045	0.042
Tenure	−0.007	0.045	0.096*	0.038	0.008	0.038
edu_1	−0.01	0.062	0.06	0.052	0.011	0.052
AIA	0.124**	0.04	0.478***	0.034	0.16***	0.039
IT			0.148***	0.034	−0.186***	0.034
RC					0.457***	0.039
R^2^	0.029		0.295		0.322	
F	2.078*		26.127***		26.942***	

Due to space limitation, only one of the test results is reported for the education background. The edu_1 refers to “general high school or secondary vocational school,” *p < 0.05, **p < 0.01, ***p < 0.001.

**TABLE 4:** Decomposition of total, direct, and mediation effects.

Path	Std. Coeff	SE	95% CI	
			LLCI	ULCI
AIA→KT	0.16	0.039	0.085	0.236
AIA→IT→KT	−0.023	0.009	−0.041	−0.007
AIA→RC→KT	0.218	0.029	0.165	0.275
AIA→IT→RC→KT	0.008	0.004	0.002	0.018

#### 4.4.2 Moderation effect testing

**FIGURE 3:**
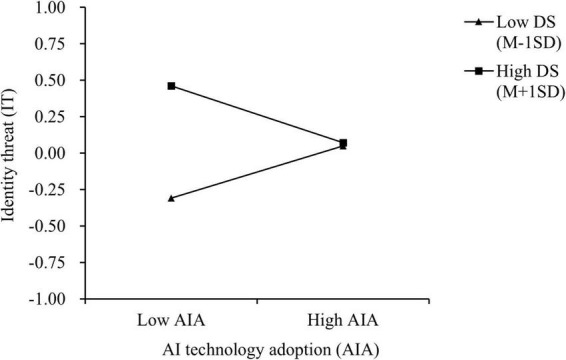
The moderating role of digital self-efficacy.

Analysis was also performed using Model 83 in the PROCESS macro version 4.2. Controlling for gender, age, monthly income, tenure, and education, the moderating role of digital self-efficacy was tested. Results (Table 5) show that the interaction term between AI technology adoption and digital self-efficacy significantly predicts identity threat (β = −0.136, *p* < 0.001), supporting Hypothesis 5. Simple slope analysis revealed that compared to older employees with low digital self-efficacy, those with high digital self-efficacy were better able to mitigate the sense of identity threat induced by AI technology adoption (Figure 3), providing further support for H5. Additional moderated mediation analysis (Table 6) indicated that for Path 1, the effect was significant in high digital self-efficacy (β = 0.027, 95% CI = [0.003, 0.06], excluding zero), and in the low digital self-efficacy (β = −0.023, 95% CI = [−0.047, −0.006], excluding zero). The difference in the mediation effect between the high and low levels of digital self-efficacy was significant, with an estimated difference of 0.051 and a 95% CI of [0.022, 0.092], excluding zero. The index was 0.025, 95% CI = [0.011, 0.046], excluding zero, confirming Hypothesis 6. Similarly, for the indirect effect of Path 2, significant effects emerged in high digital self-efficacy (β = −0.01, 95% CI = [−0.022, −0.001], excluding zero), and the low digital self-efficacy (β = 0.009, 95% CI = [0.002, 0.017], excluding zero). The difference in the mediation effect between high and low digital self-efficacy was significant, with an estimated difference of −0.018 and a 95% CI of [−0.033, −0.007], excluding zero. The Index was −0.009 (95% CI = [−0.017, −0.004], excluding zero), thus supporting Hypothesis 7.

**TABLE 5:** Analysis of moderating effects.

Variable	Identity threat			
	Std. Coeff	SE	*T*-value	95% CI
Gender	−0.032	0.04	−0.808	[−0.11,0.046]
Age	−0.013	0.041	−0.312	[−0.094,0.068]
Income	0.038	0.049	0.773	[−0.059,0.135]
Tenure	−0.004	0.044	−0.091	[−0.09,0.082]
edu_1	−0.007	0.061	−0.112	[−0.123,0.112]
AIA	−0.011	0.047	−0.233	[−0.103,0.081]
DS	0.173***	0.046	3.786	[0.083,0.263]
AIA × DS	−0.136***	0.032	−4.255	[−0.199,−0.073]
R^2^	0.076			
F	4.631***			

***p < 0.001.

**TABLE 6:** Moderated mediation effect.

Path	Moderator	Value	SE	95% CI	
				LLCI	ULCI
Path 1: AIA→IT→KT	High DS (+1SD)	0.027	0.015	0.003	0.06
	Low DS (−1SD)	−0.023	0.011	−0.047	−0.006
	Difference (high-low)	0.051	0.018	0.022	0.092
	Index value	0.025	0.009	0.011	0.046
Path 2: AIA→IT→RC→KT	High DS (+1SD)	−0.01	0.005	−0.022	−0.001
	Low DS (−1SD)	0.009	0.004	0.002	0.017
	Difference (high-low)	−0.018	0.007	−0.033	−0.007
	Index value	−0.009	0.003	−0.017	−0.004

### 4.5 Fuzzy-set qualitative comparative analysis

The aforementioned research addresses the linear mechanisms between variables, namely how AI technology adoption affects intergenerational knowledge transfer. However, it has not yet effectively examined the multiple potential pathways that may trigger high levels of intergenerational knowledge transfer. Therefore, this paper draws on the work of other scholars and employs the fsQCA method within a regression framework (Fiss et al., 2013) to interpret complex combinatorial causal relationships. This approach enables a more thorough investigation of solution pathways and the analysis of how multiple variable interactions impact the dependent variable. Consequently, this study utilizes fsQCA to examine the combinatorial configurations of antecedent conditions affecting intergenerational knowledge transfer, thereby enhancing the robustness of the findings and compensating for the limitations of linear model analysis. Based on existing empirical research, this paper selects four variables as antecedent conditions for intergenerational knowledge transfer: AI technology adoption, identity threat, relational crafting, and digital self-efficacy.

Prior to conducting the fsQCA study, variables were calibrated using three anchor points: full membership (0.95), crossover point (0.5), and full non-membership (0.05). Data points with a membership degree of exactly 0.5 were adjusted by +0.001. Following variable calibration, a single-factor necessity analysis was performed. As shown in Table 7, the consistency scores for all four factors fell below 0.9, indicating that intergenerational knowledge transfer is not determined by any single factor alone, thus necessitating a multi-factor configurational analysis.

**TABLE 7:** Result of necessary condition analysis.

Variable	KT		∼KT	
	Consistency	Coverage	Consistency	Coverage
AIA	0.74	0.735	0.593	0.573
∼AIA	0.57	0.59	0.726	0.731
IT	0.677	0.672	0.664	0.641
∼IT	0.639	0.662	0.66	0.665
RC	0.74	0.807	0.534	0.566
∼RC	0.602	0.57	0.818	0.754
DS	0.712	0.816	0.509	0.567
∼DS	0.622	0.566	0.835	0.738

#### 4.5.1 Sufficiency analysis

Configurational analysis was employed to examine the sufficiency of different combinations of multiple antecedent conditions in producing the outcome. Given the large sample size of this study, the frequency threshold was set at 4, the consistency threshold at 0.8 and the PRI threshold at 0.6 (Samuli et al., 2021). This analysis yielded three configurations that trigger intergenerational knowledge transfer and two configurations that inhibit it. These results demonstrate that multiple distinct pathways can achieve intergenerational knowledge transfer. Following the presentation format for QCA results proposed by Fiss (2011), Table 8 was constructed. As shown in Table 8, the solution consistency for intergenerational knowledge transfer is 0.852 (exceeding the 0.8 threshold). The solution coverage reaches 0.714 (surpassing the 0.5 threshold). These three causal configurations collectively explain 71.4% of the cases involving intergenerational knowledge transfer. The configuration analysis reveals the following.

**TABLE 8:** Result of configuration analysis.

Variable	KT			∼KT	
	P_1_	P_2_	P_3_	P_4_	P_5_
AIA		●	●	⊗	
IT		⊗	⊗		⊗
RC	●	●		⊗	⊗
DS	●		●	⊗	⊗
Consistency	0.877	0.918	0.916	0.836	0.858
Raw coverage	0.618	0.414	0.413	0.626	0.575
Unique coverage	0.253	0.049	0.048	0.12	0.07
Overall solution consistency	0.852			0.827	
Overall solution coverage	0.714			0.695	

● represents the core existence condition; ⊗ represents that the core condition is missing; The blank area represents whether the condition is present or missing.

Configuration P1 (intergenerational bridging type) indicates the antecedent conditions for intergenerational knowledge transfer are relational crafting and digital self-efficacy. Under this configuration: high digital self-efficacy empowers older employees with strong technological awareness and adaptive capabilities, facilitating their successful adoption of AI technology. Relational crafting provides social support resources through renewed intergenerational interaction patterns. This combination both stimulates older employees’ capability and motivation to engage in knowledge transfer and enables them to enter a resource gain spiral, thereby promoting intergenerational knowledge transfer. Crucially, this dual mechanism can overcome potential identity threats triggered by AI technology adoption and safeguard knowledge transfer, validating parts of Hypotheses 2 and 4.

Configuration P2 (technology synergy type) indicates that the antecedent conditions affecting intergenerational knowledge transfer are AI technology adoption, relational crafting, and the absence of identity threat. In this configuration, the absence of identity threat prevents the depletion of older employees’ physical and mental resources, thereby eliminating the potential negative impacts of AI technology adoption. Consequently, older employees are more inclined to invest their resources in adapting to new relational boundary conditions, which facilitates relational crafting. Furthermore, the inherent efficiency and portability of AI technology can be more effectively manifested within the organization, promoting intergenerational knowledge transfer behaviors–partially validating Hypotheses 1 and 3.

Configuration P3 (digital self-efficacy driven type) demonstrates that the antecedent conditions influencing intergenerational knowledge transfer are AI technology adoption, digital self-efficacy, and the absence of identity threat. This configuration highlights digital self-efficacy as a critical factor, where high digital self-efficacy serves as a key personal resource enabling older employees to effectively interpret and adapt to AI technology. The absence of identity threat signifies the deactivation of resource depletion pathways. Under this configuration, digital self-efficacy acts as an initiating pathway for intergenerational knowledge transfer: individuals with high digital self-efficacy can better perceive AI technology as an effective gain even in the absence of relational crafting while mitigating the negative impact of identity threat, thereby partially validating Hypothesis 4.

Based on the configurational logic, this study also identifies two additional configurations that lead to non-intergenerational knowledge transfer. Configuration P4 indicates that the absence of AI technology adoption, relational crafting, and digital self-efficacy triggers non-intergenerational knowledge transfer. Specifically, regardless of the presence of identity threat, the lack of AI technology adoption, relational crafting, and digital self-efficacy results in non-intergenerational knowledge transfer. Under this configuration, all channels for individuals to access resources are blocked. Unable to acquire new resources, individuals activate resource-protection mechanisms and cease actively pursuing intergenerational knowledge transfer, instead adopting self-defensive strategies to prevent further depletion of existing resources. Configuration P5 demonstrates that the mere absence of identity threat is insufficient to stimulate intergenerational knowledge transfer. When both digital self-efficacy and relational crafting are missing, the resource-gain pathway becomes ineffective, leading older employees to develop pessimistic expectations about future resource acquisition. Additionally, the absence of both identity threat and relational crafting indicates that the coping strategy of investing substantial resources under high job demands will also fail, resulting in non-intergenerational knowledge transfer.

## 5 Discussion

This study aims to explore the impact of AI technology adoption on intergenerational knowledge transfer among older employees. Based on JD-R and COR theories, we propose that AI technology adoption may trigger both gain and loss pathways, manifested through relational crafting and identity threat, respectively. The study also finds that employees under high job demands can better engage and acquire resources. Furthermore, digital self-efficacy, as an important individual characteristic, moderates these psychological processes. Through empirical testing, all proposed hypotheses are validated. Overall, the JD-R model provides a solid theoretical foundation for this study, and its explanatory power in technology-driven organizational change has been well validated. Moreover, by integrating the JD-R and COR theories, the study offers a more detailed explanation of older employees’ behavior from the perspective of individual resource gains and losses.

First, the findings reveal that AI technology adoption has a dual impact on intergenerational knowledge transfer, moving beyond the previous limitations of studying its isolated effects. Second, the study confirms that AI technology adoption enhances intergenerational knowledge transfer among older employees through relational crafting. Existing research suggests that AI technology can improve employees’ initiative (Chowdhury et al., 2023), thereby influencing their knowledge sharing (Shaikh et al., 2023). While knowledge transfer and knowledge sharing are distinct variables, they share similarities. Thus, the results of this study both align with and complement existing research. Third, the study uncovers a pathway through which AI technology adoption inhibits intergenerational knowledge transfer via identity threat. Previous scholars have demonstrated that AI technology adoption may trigger perceived unemployment risks, leading to job insecurity (Koo et al., 2021). This supports the notion that AI technology adoption can indeed induce negative effects, consistent with the conclusions of this study. Fourth, the study finds that older employees experiencing high identity threat (a high job demand) proactively engage in relational crafting to counter potential resource loss and acquire new resources, thereby promoting intergenerational knowledge transfer. Extensive prior research has confirmed that high job demands can trigger active coping strategies in specific contexts (Tadić et al., 2015), which aligns with the findings of this study. Fifth, the study demonstrates that digital self-efficacy significantly moderates the relationship between AI technology adoption and identity threat, as well as the associated mediating pathways. Older employees with high digital self-efficacy are less likely to perceive AI technology as a threat, reducing the likelihood of decreased intergenerational knowledge transfer and their reliance on the threat-coping pathway of identity threat and relational crafting. This moderating effect highlights the importance of individual characteristics in responding to AI-driven changes, an aspect underexplored in previous research. Thus, this study provides deeper insights into how individual characteristics shape the impact of AI technology adoption on older employees. Sixth, using fsQCA, the study examines the multiple concurrent factors and complex mechanisms of intergenerational knowledge transfer. The results reveal that the pathways to intergenerational knowledge transfer are not singular. Three distinct configurations lead to high levels of intergenerational knowledge transfer: intergenerational bridging type, technology synergy type, and digital self-driven type. This approach addresses the limitations of previous linear models and offers a more comprehensive understanding of the psychological and behavioral mechanisms of older employees in the digital-intelligent era from a configurational perspective.

### 5.1 Theoretical implications

This study makes several contributions. First, while academic research has explored the definition, measurement, antecedents, and consequences of AI technology adoption, it has predominantly focused on antecedents rather than consequences. Moreover, previous studies frequently employed TAM to examine antecedents from external or internal environmental perspectives (Ashfaq et al., 2020). In contrast, this study uses the JD–R model and COR theory to explain consequences of AI technology adoption from the perspective of resource gains and losses. This shifts the research focus from “whether to adopt” to “how adoption influences,” thereby expanding the theoretical framework of outcome variables related to AI technology adoption. Existing limited research on consequences has primarily verified positive or negative effects on specific variables but has overlooked the diverse and complex impacts on individuals. By constructing a moderated sequential mediation model that concurrently incorporates both positive and negative effects, this study elucidates multiple mechanisms through which AI technology adoption influences intergenerational knowledge transfer, providing a more comprehensive explanation of coexisting positive and negative effects.

Second, whereas prior work often frames older employees as “digital immigrants” and emphasizes obstacles and negative psychological effects in adopting AI (Román-García et al., 2016), this study reveals a more positive and dynamic process. Previous scholars have typically treated identity threat as a negative factor (Milad et al., 2022). Here, AI technology adoption can significantly promote intergenerational knowledge transfer among older employees through the sequential mediation of identity threat and relational crafting. Older employees can actively reconstruct social relationships, seek new value orientations, establish collaborative relations with younger employees, better adapt to technological changes, and achieve knowledge inheritance. This finding validates the coping hypothesis in JD–R theory and the mechanism of “positive coping in resource-loss situations” in COR theory, offering a more nuanced perspective on older employees’ psychology and behavior in the digital era. It also extends the application contexts of both JD-R and COR theories, offering a more scientifically grounded explanation for understanding the psychological and behavioral patterns of older employees in the digital–intelligent era.

Third, using a configurational approach, this study identifies three high–intergenerational-knowledge-transfer pathways, demonstrating that knowledge transfer results from the interplay of multiple factors. While existing research on consequences of AI technology adoption predominantly follows linear analytical frameworks, this study shows that intergenerational knowledge transfer is not determined by singular linear relationships. Moreover, prior studies have mainly examined mechanisms from the perspective of “presence” while neglecting the “absence” perspective, which limits understanding of the mechanisms underlying intergenerational knowledge transfer under AI technology adoption. The two antecedent configurations leading to non-intergenerational knowledge transfer provide valuable supplementation and respond to causal asymmetry in complex organizational contexts. This demonstrates that pathways leading to the presence and absence of outcomes are not simple mirror opposites, offering a more comprehensive theoretical account of behavioral heterogeneity under AI technology adoption.

### 5.2 Management insights

First, organizations should prioritize investments in enhancing older employees’ digital self-efficacy to mitigate potential identity threat. The moderating effect analysis of this study indicates that digital self-efficacy is key personal resource for buffering the negative psychological impact of AI technology adoption (Maran et al., 2022). Therefore, managers should not only provide basic AI technical training but also actively boost older employees’ confidence in using AI technologies to bridge the digital divide. By sharing success stories and conducting simulated practices, organizations can effectively improve older employees’ digital self-efficacy, thereby alleviating identity threat at its root and promoting sustainable organizational development.

Second, organizations need to proactively create opportunities for older employees to engage in relational crafting. The empirical results of this study show that relational crafting, as a critical job resource, not only stimulates older employees’ intergenerational knowledge transfer but also mitigates resource depletion caused by identity threat. Thus, managers should regularly organize intergenerational exchange sessions and encourage collaboration between older and younger employees in AI application. This helps older employees rebuild their social networks and demonstrate the value of their experience (Geldenhuys et al., 2020).

Finally, managers should adopt an integrated, configuration-based approach in their practices. The fsQCA results of this study reveal that there are multiple equivalent pathways to achieving high levels of intergenerational knowledge transfer. Therefore, in management practice, it is essential to assess different types of older employees and implement tailored strategies. For older employees with high digital self-efficacy, AI technology can be mainly introduced; for those lacking social interaction, efforts can be made to strengthen relational crafting. The core objective is to reduce potential identity threats triggered by AI technology and foster an inclusive corporate technological culture.

In summary, as organizations introduce AI technology, they must understand the paths of technological evolution. By enhancing digital self-efficacy, reducing identity threat, and promoting relational crafting, organizations can build an inclusive corporate technological culture that systematically facilitates effective intergenerational knowledge transfer among older employees, thereby achieving sustainable development for both the enterprise and its older workforce.

### 5.3 Shortcomings and prospects

This study also has several limitations. First, all data were self-reported by employees, which may introduce common method bias. Moreover, AI implementation in organizations is a long-term process, and its impact on employees varies across phases. Future research could adopt multi-wave, multi-source designs with repeated measurements and longitudinal tracking of AI technology adoption stages to mitigate such biases and clarify outcome dynamics at different phases. Second, while focusing on how older employees’ personality traits and psychological states influence intergenerational knowledge transfer under AI technology adoption, this study may overlook organizational and contextual differences across cultures. Finally, the depth of the configuration analysis in this study can be further improved, especially regarding the pathway from high job demands to individual resource investment and empirical characterization of the sequential mediation path.

## Data Availability

The raw data supporting the conclusions of this article will be made available by the authors, without undue reservation.
